# Dehydrins Impart Protection against Oxidative Stress in Transgenic Tobacco Plants

**DOI:** 10.3389/fpls.2018.00136

**Published:** 2018-02-14

**Authors:** Tanmoy Halder, Gouranga Upadhyaya, Chandra Basak, Arup Das, Chandrima Chakraborty, Sudipta Ray

**Affiliations:** Plant Functional Genomics Laboratory, Centre of Advanced Study, Department of Botany, University of Calcutta, Kolkata, India

**Keywords:** dehydrin, oxidative stress, methyl viologen, electron transport chain, protoplast

## Abstract

Environmental stresses generate reactive oxygen species (ROS) which might be detrimental to the plants when produced in an uncontrolled way. However, the plants ameliorate such stresses by synthesizing antioxidants and enzymes responsible for the dismutation of ROS. Additionally, the dehydrins were also able to protect the inactivation of the enzyme lactate dehydrogenase against hydroxyl radicals (OH^⋅^) generated during Fenton’s reaction. *SbDhn1* and *SbDhn2* overexpressing transgenic tobacco plants were able to protect against oxidative damage. Transgenic tobacco lines showed better photosynthetic efficiency along with high chlorophyll content, soluble sugar and proline. However, the malonyl dialdehyde (MDA) content was significantly lower in transgenic lines. Experimental evidence demonstrates the protective effect of dehydrins on electron transport chain in isolated chloroplast upon methyl viologen (MV) treatment. The transgenic tobacco plants showed significantly lower superoxide radical generation (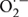) upon MV treatment. The accumulation of the H_2_O_2_ was also lower in the transgenic plants. Furthermore, in the transgenic plants the expression of ROS scavenging enzymes was higher compared to non-transformed (NT) or vector transformed (VT) plants. Taken together these data, during oxidative stress dehydrins function by scavenging the (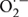) directly and also by rendering protection to the enzymes responsible for the dismutation of (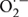) thereby significantly reducing the amount of hydrogen peroxides formed. Increase in proline content along with other antioxidants might also play a significant role in stress amelioration. Dehydrins thus function co-operatively with other protective mechanisms under oxidative stress conditions rendering protection in stress environment.

## Introduction

Several environmental situations such as drought, salinity, temperature variation are the major stress factors, which hamper plant growth throughout their life cycle. The primary stress factors like drought, salinity, high temperature may further culminate into oxidative stress causing severe cellular damage (Foyer and Mullineaux, 1994; Van Breusegem et al., 1999; Mittler, 2002). The ROS generated in cells produce oxidative stress due to the formation of ^1^O_2_, 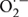, H_2_O_2_, and the hydroxyl radicals (HO^⋅^). However, tolerance to such damaging conditions may be achieved by effective scavenging mechanisms for the excessively accumulated ROS.

The cellular protection and damage repair mechanism work together to increase the stability of the proteins and biomolecules and preventing protein aggregation. Proteins stability and prevention of aggregation can be achieved by accumulating molecular chaperones as well as highly hydrophilic proteins like dehydrins, a group II LEA proteins (Konrad and Bar-Zvi, 2008; Halder et al., 2016). In general, all dehydrins contain at least a copy of K segment (EKKGIMDKIKEKLPG) usually present in the C-terminus of the protein. Along with the K segment, dehydrin may possess some other conserved motifs viz. Y segment (T/VDEYGNP), S segment (a motif rich in Ser residues) and randomly present Φ-segment (Close, 1997). The number and distribution pattern of the K, Y, and S motifs define the different sub-classes of dehydrins viz. K_n_S, K_n_, Y_n_SK_n_, Y_n_K_n_, SK_n_. Numerous studies have been documented suggesting different molecular functions of the protein. These include cryoprotection (Agarwal et al., 2017), protecting cell membrane from lipid peroxidation (LPO), scavenging ROS and metal binding activity. Radical scavenging activity and oxidative modification of CuCOR19 and AtHIRD11 were earlier reported by Hara et al. (2004, 2013). Earlier report by Hara et al. (2004) showed that CuCOR19 (a K_n_S type dehydrin from *Citrus*) was capable of scavenging the hydroxyl radical generated by the Fe^2++^/H_2_O_2_ system and peroxyl radical generated from 2, 2′-azobis (2-amidinopropane). Earlier reports also suggested the protective role of dehydrins against oxidative damage caused by ROS and metal ions (Zhang et al., 2006). Earlier report of a K_n_S type dehydrin was shown to bind metal ions (Hara et al., 2005). Sequestration of the metal ions inhibits the production of hydroxyl radical (OH^⋅^) through Fenton’s reaction (Genaro-Mattos et al., 2015). The hydroxyl radicals are highly deleterious to cellular components; therefore, their levels are kept at a minimal level as compared to superoxides and H_2_O_2_. Enhanced tolerance toward oxidative as well as salt stress was achieved by over-expressing MusaWRKY71 in banana plants leading to better photosynthetic efficiency (*F*v/*F*m) and lower membrane damage (Shekhawat and Ganapathi, 2013). Dehydrin can protect the cell membranes against LPO during oxidative stress condition (Hara et al., 2003). SK_n_ type dehydrins (Danyluk et al., 1998; Yang et al., 2014) were found near the plasma membrane. The *in vitro* association of dehydrins to membranes has been shown by several researchers (Ismail et al., 1999; Hara et al., 2001; Koag et al., 2003, 2009; Soulages et al., 2003). Their work suggested that dehydrins gain α-helical structure in the presence of negatively charged liposomes and micelles. It was shown previously that the K-segment is responsible for binding to membranes (Koag et al., 2009). It has been proposed by Eriksson et al. (2011) that the histidine residues located on either side of the K-segments in LTI30 help to modulate membrane binding. However, all dehydrin K-segments are not flanked by His residues and even the K-peptide alone has been shown to bind vesicles (Koag et al., 2009) which clearly indicate that occurrence of these histidine residues near the K-segments are not critical for membrane binding activity for other dehydrins. Depending on the lipid compositions mimicking the plasma membrane, mitochondrial membrane and chloroplast membrane, *Thellungiella salsuginea* dehydrin 1 (TsDHN-1) showed difference in type and amount of structural change (Rahman et al., 2010, 2013). The membranes mimicking the chloroplast are considerably different from others, as they primarily consist of the galactolipids monogalactosyldiacylglycerol (MGDG) and digalactosyldiacylglycerol (DGDG), which are neutral. The binding of dehydrins to plant outer and organellar membranes should therefore impart protection to the plants under stress conditions. The major sources of ROS are organelles such as chloroplasts, mitochondria or microbodies which have a highly oxidizing metabolic activity or having an intense rate of electron flow (Apel and Hirt, 2004). The chloroplast and peroxisomes are the two major organelles contributing to the oxidative load in plant cells during environmental stress. In order to ward off the oxidative damage in plants, a number of mechanisms orchestra together to handle the oxidative stress conditions. This is generally achieved by production of antioxidants and radical scavengers along with an increase in enzymes responding to dismutation of free radicals. The imbalance between the production of ROS and its scavenging mechanisms may initiate uncontrolled oxidative cascades.

The enhancement of ROS, under stressed condition, acts as an alarm bell for triggering the defense response pathway genes where H_2_O_2_ acts as a secondary messenger in the signal transduction pathway (Orozco-Cárdenas et al., 2001). A versatile and cooperative antioxidant system in the cell keeps the enhanced ROS production under tight control. An array of enzymatic and non-enzymatic antioxidants functions as an efficient system to keep the ROS level under control. Among the ROS the superoxides are primarily produced in large amount upon encountering oxidative stress (Apel and Hirt, 2004). SOD serves as a major scavenging enzyme in dismutating superoxides to O_2_ and H_2_O_2_; however, this only converts the ROS from its one form to other. The H_2_O_2_ thus produced in this reaction can attack the thiol proteins. CAT and APX are the other major enzymatic proteins that can convert the H_2_O_2_ into water and oxygen. Previous reports also showed an increase in SOD, POX, and CAT activity in overexpressed dehydrin plants which showed enhanced tolerance under oxidative stress conditions (Liu et al., 2017).

Here in this current study two dehydrin genes, *SbDhn1* (Accession no. KT865881) and *SbDhn2* (Accession no. KT780443) were analyzed under oxidative stress conditions. *SbDhn1* (YSK_2_ type) and *SbDhn2* (SK_3_ type) were previously isolated and characterized from *Sorghum bicolor* (Halder et al., 2016, 2017). Previously we reported that *SbDhn1* was found to play a protective role under high temperature and osmotic stress when over expressed in tobacco plants. The ease of *Agrobacterium* mediated transformation, and optimized tissue culture media make it suitable for use as a model plant. Previous data also showed that *SbDhn2* possessed metal binding as well as radical scavenging activity (Halder et al., 2016). Here, we compare the scavenging activity between the two dehydrins (SbDHN1 and SbDHN2). The metal sequestration activity of some dehydrins could prevent the (OH⋅) generation by sequestering the metal ions required in Fenton’s reaction. In order to elucidate the role of dehydrin genes during oxidative stress conditions, transgenic tobacco plants overexpressing dehydrins were subjected to MV. The transgenic lines showed better photosynthetic efficiency under oxidative stress conditions. Here, we provide direct evidence for the protection rendered by dehydrins during oxidative stress conditions to isolated chloroplast when added externally and also when synthesized *in planta*. The membrane binding activity of the dehydrins might be responsible for such protection. Dehydrins could protect the plants directly by scavenging the ROS or rendering overall protective effect to the enzymes responsible for dismutation of free radicals. Thus overexpression of dehydrin genes in transgenic tobacco plants showed better physiological conditions as compared to that of NT and VT plants.

## Materials and Methods

### Cloning, Expression, and Purification of *SbDhn1* and *SbDhn2*

The cloning, expression, and purification of the two proteins *SbDhn1* and *SbDhn2* were carried out as described previously by Halder et al. (2016, 2017). The dehydrin genes were amplified by gene specific primers (Supplementary Table S1) from *Sorghum bicolor* and cloned in pGEMT easy vector. The CDS was subcloned in bacterial expression vector at NdeI and XhoI sites. The expression was induced by 1mM IPTG at 37°C at 180 rpm in Rosetta (DE3) pLysS cells. The expressed recombinant protein was purified by affinity column chromatography with the help of His tagged and GST tagged fusion protein, which was subsequently digested by Factor Xa. After affinity purification, the purified protein was confirmed by immunoblotting with anti-His antibody and HRP conjugated secondary antibody.

### Generation of *SbDhn1* and *SbDhn2* Overexpressing Tobacco Lines

The *SbDhn1* transformed plants were raised as described previously by Halder et al. (2017). The *SbDhn2* gene was placed under the control of CaMV35S promoter and cloned in plant expression vector. The chimeric plant expression cassette was then mobilized in *Agrobacterium tumefaciens* strain LBA4404 by freeze-thaw method. *Agrobacterium*-mediated tobacco leaf disc (*Nicotiana tabacum*; variety SR) transformation was carried out as described by Horsch et al. (1985). The putative transgenic plants were analyzed by PCR amplification of dehydrin (*SbDhn1* or *SbDhn2*) and hygromycin resistance genes (*hpt*). Details of the oligonucleotide primers used in this study are given in Supplementary Table S1.

### Effect of Dehydrins on LDH Activity under Oxidative Stress

In order to evaluate the protective activity of the SbDHN1 and SbDHN2 protein under oxidative stress condition, LDH protection assay was performed as described previously by Halder et al. (2016). The effect of free radicals on LDH was determined by incubating 0.05 U of LDH with 0.1 mM FeSO_4_ and 1 mM H_2_O_2_ in absence or presence of SbDHN1 or SbDHN2 or BSA at different concentrations ranging from 200 ng/μl to 1 μg/μl, for 10 min at 25°C. Following incubation, LDH activity was measured in 1 ml of reaction mix containing 1.1 mM pyruvic acid and 0.13 mM NADH. Oxidation of NADH was calculated from the reading obtained at A_340_ for 5 min in a spectrophotometer (Jasco, V-730) during the linear reaction rate. *N*-Acetyl-L-cysteine (50 μM) a known radical scavenger was used as a positive control to compare the radical scavenging activity of the dehydrin proteins. The effect of either FeSO_4_ or H_2_O_2_ alone on the LDH activity was also evaluated. In order to do so same amount of LDH was incubated with different concentration of FeSO_4_ ranging from 0.01 mM to 0.25 mM or different concentration of H_2_O_2_ ranging from 0.1 mM to 2.5 mM for 10 min at 25°C. Following incubation, the LDH activity was measured as mentioned above. The protective effect of dehydrins on the LDH in presence of either FeSO_4_ or H_2_O_2_ was also evaluated. Same amount of LDH was incubated with either 0.1 mM FeSO_4_ or 1 mM H_2_O_2_ in presence or absent of 1 μg/μl of SbDHN1or SbDHN2 or BSA. Following incubation, the LDH activity was measured.

### Effect of Oxidative Stress on Transgenic Plants

For evaluating the effect of the MV on transgenic plants, leaf disc assay was carried out. Leaf discs (1 cm diameter) were punched from the third and fourth fully expanded leaves of transgenic plants (*SbDhn1* or *SbDhn2* lines), NT, as well as VT lines. Leaf discs were collected from three different lines for *SbDhn1* or *SbDhn2* plants and the assay was carried out in triplicate. The leaf discs were then placed in half MS media (Murashige and Skoog, 1962) containing 20 μM MV with the abaxial side of the leaf facing the media. The leaf discs were incubated for 5 days under continuous illumination at 22°C temperature and 70% relative humidity. The leaf discs from a NT plant were placed in half MS medium without MV which served as a control. Photographs were taken at 0 day and 5 days after incubation.

In order to evaluate the effect of MV on whole plants, transgenic plants overexpressing dehydrin genes (*SbDhn1*: Lines and *SbDhn2*: Lines), NT and VT plants were grown under normal culture conditions (25°C temperature and 70% relative humidity with 16 h photoperiod) for 21 days. The plants were then transferred to MS basal media (Murashige and Skoog, 1962) containing 50 μM MV and incubated for 5 days under continuous illumination at 22°C temperature and 70% relative humidity. The plants were photographed before and after the stress treatment. The whole experiment was repeated three times.

### Estimation of Proline Content

Proline content was estimated by using the method described by Ábrahám et al. (2010). Acidic ninhydrin reagent was used to estimate the total proline content of the plants. The reagent was prepared by dissolving 2.5 g of ninhydrin in 100 ml of the solution containing glacial acetic acid: distilled water: 85% orthophosphoric acid at a ratio of 6:3:1. One hundred milligrams of leaf samples were homogenized in 1 ml of 3% (w/v) aqueous sulfosalicylic acid solution and centrifuged at 12,000 × *g* for 15 min at 4°C. Following incubation, 1 ml of supernatant was collected and mixed with equal volume of glacial acetic acid and ninhydrin reagent and kept in a boiling water bath for 1 h. The reaction was stopped by incubating the reaction in a water bath at room temperature (21°C) for 5 min. The absorbance was measured immediately at 546 nm using Jasco V730 IRM spectrophotometer. Total proline content was estimated by using a proline standard curve as a reference and expressed in mg/g fresh tissue.

### Determination of the Malonyldialdehyde (MDA) Content

Malonyl dialdehyde content was estimated as per the method of Baryla et al. (2000). For quantification of MDA content in stressed and unstressed transgenic, NT, and VT tobacco plants, approximately, 100 mg of leaf material was extracted in 1 ml 0.1% (w/v) TCA solution. The homogenates were centrifuged at 12,000 × *g* for 15 min at 4°C. Five hundred microliters of supernatant was then mixed with 1 ml 0.5% TBA solution (500 mg TBA dissolved in 100 ml of 20% TCA). Then the reaction mixture was placed in a boiling water bath for 1 h and immediately kept on ice for 5 min. The absorption of the samples was measured at 532 nm, after centrifugation at 10,000 × *g* for 10 min. The data were calculated from the extinction coefficient of MDA (155 mM^-1^ cm^-1^) after subtracting the value of non-specific absorption at 600 nm.

### Total Chlorophyll Content

Total chlorophyll content was measured spectrophotometrically according to Arnon (1949). Briefly, 100 mg of leaf were crushed in 2 ml of 80% acetone followed by centrifugation at 10,000 × *g* for 10 min at 25°C. The clear supernatant was collected and absorbance at 645 nm and 663 nm were recorded. The total chlorophyll content was calculated using the formula given below:

$$Total\,\;chlorophyll=\frac{\left[20.2(D645)+8.02\left(D663\right)V\right]}{(1000)W}$$

[Where, D, optical density value; V, final volume of 80% alkaline acetone; W, fresh weight in grams of tissue extract]. The total chlorophyll content was estimated in the term of mg/g of fresh tissue.

### Estimation of Total Soluble Sugar

Total soluble sugar content was estimated according to Irigoyen et al. (1992) with some minor modifications. Leaf samples (100 mg) were extracted in 1 ml of 95% ethanol followed by centrifugation at 13,000 × *g* for 10 min at 25°C. After centrifugation, 100 μl supernatant was mixed with 1 ml anthrone solution (15 mg anthrone dissolved in 10 ml of 72% H_2_SO_4_), and incubated for 10 min in water bath at 90–95°C. Following incubation, the samples were cooled and the absorbance was measured at 625 nm using Jasco V730 IRM spectrophotometer. Total soluble sugar was estimated by using glucose as a standard reference and expressed in mg/g fresh weight of leaves.

### Measurement of Photosynthetic Efficiency

The photosynthetic efficiency of stressed (*SbDhn1*, *SbDhn2*, NT and VT plants) as well as unstressed NT plants were analyzed using a portable Chlorophyll Fluorimeter (Handy PEA, Hansatech Instruments, Ltd., Norfolk, United Kingdom) by estimating chlorophyll fluorescence. The distal extremities of the central vein of the leaf blade of the third and fifth leaves (counted from the top) were measured. Firstly, the sample area to be analyzed was covered by leaf clips for dark adaptation. Three leaf clips were attached to the leaf to maintain a particular area under the dark condition for 30 min so that the reaction centers acquire the open condition (oxidized Qa) (Bolhar-Nordenkampf et al., 1989). After dark adjustment, illumination was supplied by an array of six LEDs emitting red light, optically filtered to a peak wavelength of 650 nm at a maximum intensity of up to 3,500 μmol m^-2^ s^-1^ on the surface of leaf (4 mm in diameter) placed in the clips. Leaf model was generated from JIP-test parameters by using the Biolyzer software (Discoverer: Ronald Maldonado Rodriguez) and the following variables were measured and calculated; (1) ABS/CS_m_ ratio: the total number of photons absorbed by an excited PS II cross section. (2) TR_0_/CS_m_: the maximal trapping rate of an exciton that will lead to Qa reduction measured over a cross-section of active and inactive RCs (reaction centers), (3) DI_0_/CS_m_: The dissipation of untrapped excitation energy per excited cross-section. Dissipation occurs as heat, fluorescence and energy transfer to other systems. It is influenced by the ratio of active/inactive RCs, (4) Electron transport in a PS II cross section (ET_0_/CS_m_): deals with the reoxidation of reduced Qa via electron transport over a cross-section of active and inactive RCs (Force et al., 2003).

### Isolation of Chloroplast and Electron Transport Chain Assay

Intact chloroplasts were isolated from tobacco plants as described by Nakano and Asada (1981) with some minor modifications. Briefly, 50 mM phosphate buffer pH 7.0, containing 0.4 M sucrose and 10 mM KCl was used as CIB. Plant material was homogenized by using mixer grinder; filtered through 50 μm mesh cloth and the filtrate was centrifuged at 200 × *g* for 5 min at room temperature. The supernatant was collected and again centrifuged at 1,500 × *g* at room temperature for 7 min to pellet down the chloroplasts. The supernatant was discarded and the pellet containing the chloroplast was redissolved in CIB. Intact chloroplasts were then purified through sucrose gradient. The number of intact chloroplasts was determined by using haemocytometer and an equal amount of chloroplast was used for electron transport assay.

The isolated chloroplasts were suspended in assay buffer containing (50 mM phosphate buffer pH 7.0, 0.05 M KCl) in presence or absence of SbDHN1 or SbDHN2 protein at a concentration of 2 μg/ml. One millimolar MV was used as an electron acceptor and generation of oxidative stress and 0.05 μg/ml DCPIP was used as a reducing agent. The samples were illuminated for 5 min and absorbance was measured at 604 nm. The reaction mixture without any protein was used as a negative control and with 2 μg/ml of BSA was used as a positive control. The 100% activity was the activity shown by the isolated chloroplast in absence of MV. The data represented here shows the percentage of protection of the protein against oxidative stress.

The chloroplasts were also isolated from NT, VT, *SbDhn1*, and *SbDhn2* transformed lines. The experimental setup was same as described previously. In these experiments, no protein was added externally. The over expressed lines of *SbDhn1* and *SbDhn*2 were used to measure the level of protection imparted by the dehydrin proteins during oxidative stress. The chloroplasts isolated from NT plant without MV served as a control in this experiment. Absorbance was measured at 604 nm after incubating for 5 min under light condition.

### Superoxide Radical Generation in Tobacco Protoplast

Protoplasts were isolated from leaf tissue of transgenic, NT and VT plants according to Yoo et al. (2007) with few modifications. One hundred milligrams of leaf samples were cut into 0.5–1 sq.mm leaf strips excluding the midrib using a sharp blade without crushing the tissue at the cutting edges. Leaf strips were digested with freshly prepared enzyme solution (20 mM MES, pH 5.7 containing 0.5% (w/v) cellulase ‘onozuka’ R10, 0.17% (w/v) macerozyme ‘onozuka’ R10, 0.4 M mannitol and 20 mM KCl, 10 mM CaCl_2_, and 0.1% BSA). The digestion was carried out in dark for 12 h at room temperature with mild shaking. Protoplast solution was then diluted with an equal volume of wash solution (2 mM MES pH 5.7 containing 154 mM NaCl, 125 mM CaCl_2_, and 5 mM KCl) and filtered through a 100 μm nylon mesh to remove undigested tissue. Protoplasts were then centrifuged at 100 × *g* at room temperature for 2 min and the supernatant was removed. Protoplasts were washed twice with wash solution and finally dissolved in 1X PBS (pH 7.4) supplemented with 7.2% mannitol for further use.

For evaluating the superoxide production in isolated protoplasts from NT, VT, and transformed plants under oxidative stress condition, 100 μl aliquots were treated with MV at a final concentration of 10 mM and incubated for 15 min in presence of light. Staining was done with DHE (Abcam ab145360) in dark and fluorescence micrographs were taken using fluorescence microscope with excitation maxima 392 nm and emission maxima 410 nm. The fluorescence was also recorded in a spectrofluorimeter (HITACHI Fluorescence Spectrophotometer F-7000) at λ_ex_ = 392 nm and λ_em_ = 410 nm with a slit width of 1 nm, at 25°C.

Superoxide production in the transgenic, NT and VT plants was also observed when whole plants were subjected to oxidative stress treatment. Protoplasts were isolated from leaves of transgenic, NT and VT plants which were subjected to 50 μM MV stress for 5 days and stained with DHE and photographed as described previously.

### 3, 3′-Diaminobenzidine (DAB) Staining of Tobacco Plants

Staining of tobacco leaves from *SbDhn1*, *SbDhn2*, VT and NT plants was done according to Daudi and O’Brien (2012). Fifty milligrams of DAB was dissolved in 47.5 ml of sterile H_2_O, the pH was adjusted to 3.0 by using 0.2 M HCl. 0.05% v/v Tween-20 and 5% 200 mM Na_2_HPO_4_ were added to make a total volume of 50 ml. Second fully expanded leaves of NT, VT and *SbDhn1* and *SbDhn2* transgenic plant after 5 days of MV treatment were dipped in DAB solution and placed on a shaker for 4–5 h at 90–100 rpm shaking speed at the dark condition. After incubation, the DAB buffer was replaced with bleaching solution (ethanol:glycerol:acetic acid in a ratio of 3:1:1) and placed in a water bath (90°C) for 15 min. Bleaching solution was changed twice and after cooling, the leaves were photographed.

### Enzyme Assay for SOD, POX, APX, and CAT

The activity of SOD was measured as described by Liang et al. (2011). Approximately 100 mg of leaf tissue from stressed and unstressed plants (*SbDhn1*, *SbDhn2*, VT and NT) were homogenized with 50 mM sodium phosphate buffer pH 6.8 in a cold condition. The extract was centrifuged at 12,000 × *g* at 4°C for 10 min and the supernatant was collected. The reaction mixture was prepared by addition of 100 μl of the extracts to 13 mM methionine, 75 μM nitroblue tetrazolium and 0.1 μM disodium ethylenediamine tetraacetate in 50 mM potassium phosphate buffer (pH 7.8). The reaction was initiated under 4000 Lx candescent lamp by the addition of riboflavin (1.3 μM) to the mixture. The rate of absorbance was recorded at 560 nm for 30 min. One enzyme unit (U) was defined as the amount of enzyme required for inhibition of 50% NBT.

Specific activity of enzyme POX was carried out by using the method of Malik and Singh (1980) with some modification. Crude POX extracts were prepared by grinding 100 mg of leaf tissue from stressed as well as unstressed plants (*SbDhn1*, *SbDhn2*, VT and NT) in 1 ml of 100 mM phosphate buffer pH 7.0 followed by centrifugation at 12,000 × *g* at 4°C for 10 min. Reaction mixture was prepared by using 0.1 ml enzyme extract, 900 μl of the buffer solution containing 20 mM 0.05 ml guaiacol solution and 0.03 ml H_2_O_2_ (0.042% = 12.3 mM). The time required for the optical density to be increased by 0.1 (Δt) was recorded at 436 nm and the enzyme activity was then calculated using the formula, enzyme units g^-1^ f wt = [500/Δt] × [1/1000] × [TV/VU] × [1/f wt]; Δt, time change in minute; TV, total volume of the extract (ml); VU, volume used (ml); f wt, weight of the fresh leaf tissue (g). The enzyme activity was expressed at U/mg protein.

Ascorbate peroxidase assay was carried out according to the method of Nakano and Asada (1981). Leaf tissues were collected from stressed and unstressed plants of *SbDhn1*, *SbDhn2*, VT and NT plants. They were crushed in 50 mM potassium phosphate buffer (pH 7.2) containing 1 mM ascorbate. Extracts were centrifuged at 12,000 × *g* for 10 min at 4°C and the supernatant was collected. The reaction mixture was prepared by addition of 100 μl of extract in 1 ml of extraction buffer containing 0.5 mM ascorbic acid, 0.1 mM EDTA and 0.1 mM H_2_O_2_. The reaction was carried out for 5 min at 25°C. Oxidation of ascorbic acid was examined by decrease in absorbance at 290 nm. One unit of APX was referred as the amount of enzyme oxidizing 1 μmole of ascorbic acid per minute. The enzyme activity was expressed at U/mg protein.

Catalase activity was determined spectrophotometrically following the method of Cakmak and Horst (1991). One hundred milligrams of leaf tissue was collected from transgenic plants (*SbDhn1*, *SbDhn2*), VT and NT plants subjected to MV stress and also from unstressed condition. The samples were homogenized with liquid nitrogen to a fine powder. The crushed samples were then suspended in 1 ml of 50mM sodium phosphate buffer, pH-7.0. The extract was centrifuged at 12000 × *g* at 4°C for 10 min. The reaction mixture was prepared by mixing 100μl of the enzyme extract and 50μl of H_2_O_2_ (0.3%) and volume was made up to 1ml by addition of phosphate buffer. The decrease in absorbance was recorded through time course measurement for 3 min at a wavelength of 240nm by Jasco V730 IRM spectrophotometer. CAT activity was expressed as U/mg of protein using the molar extinction coefficient 𝜖 = 43.1M^-1^cm^-1^.

### qRT-PCR Analysis for ROS Scavenging Enzymes during Oxidative Stress

The transcripts for the genes coding for ROS scavenging enzymes like SOD (XM_016612374.1), POX (D11396.1), APX (U15933.1), and CAT (U07627.1) were analyzed in transgenic tobacco plants under oxidative stress condition. Quantitative Real-time PCR (qRT-PCR) analysis was carried out with actin gene serving as an internal control for normalization. The primer sequences used for the real-time experiment were designed by Primer3 software and listed in Supplementary Table S1. The qRT-PCR was carried out using the CFX96 (Bio-Rad, United States) and SYBR Green Premix (Bio-Rad, United States). The PCR cycling condition was as follows: 95°C for 1 min, followed by 40 cycles of 95°C for 10 s and 60°C for 20 s. In order to verify the primer specificity a melting curve analysis was performed after 40 cycles. The relative expression level was calculated using the 2^-ΔΔ*C*T^ method, in which CT indicates cycle threshold.

### Statistical Analysis

All experiments were carried out in triplicate and repeated at least three times. Data shown are illustrative of at least three independent experiments. All the data were statistically analyzed using Student’s *t*-test (*P* < 0.01).

## Result

### Both SbDHN1 and SbDHN2 Were Able to Protect the Enzyme Lactate Dehydrogenase under Oxidative Stress Condition

**FIGURE 1:**
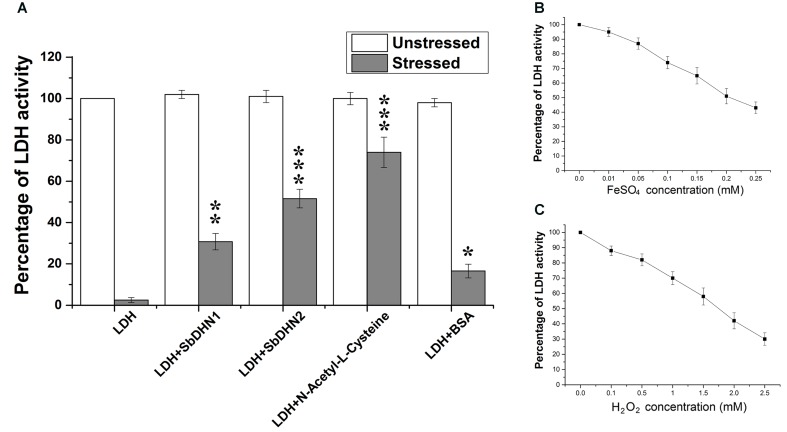
Comparative analysis of protection of LDH activity with two different dehydrins from *Sorghum bicolor* SbDHN1 and SbDHN2. **(A)** LDH was subjected to oxidative stress generated by Fenton’s reaction in presence and absence of SbDHN1, SbDHN2, *N*-acetyl-L-cysteine, and BSA. **(B)** Effect of FeSO_4_ alone on LDH activity in a concentration dependent manner. **(C)** Effect of H_2_O_2_ alone on LDH activity in a concentration dependent manner. Error bar represents ± SEM from at least three replicates. All data were statistically analyzed using Student’s *t*-test (^∗∗∗^*P* < 0.0005, ^∗∗^*P* < 0.005 and ^∗^*P* < 0.05). Data shown are illustrations of at least three independent experiments.

The enzyme LDH completely loses its activity when treated with FeSO_4_ and H_2_O_2_. Transition metal ions in their lower oxidation states like Fe^2+^ in presence of H_2_O_2_ lead to the production of hydroxyl radical (^⋅^OH). Therefore in presence of ROS like (^⋅^OH) the enzyme LDH losses it activity completely. However, more than 50% activity was found to be retained by the enzyme when it was incubated in presence of Sorghum dehydrin (SbDHN2) whereas only 30% activity was retained in presence of SbDHN1 when used at a concentration of 1 μg/μl (**Figure 1A**). The dehydrins were able to protect the LDH activity in a dose dependant manner; where the higher concentration of protein showed greater protective effect, although, after a certain concentration of the dehydrins, this protective effect reached a saturation point (Supplementary Figure S1A). The results were compared with respect to other known protectants like *N*-acetyl-L-cysteine. Using equimolar amount of the proteins (SbDHN1, SbDHN2, and BSA) for LDH protection assay, SbDHN2 was able to scavenge the hydroxyl radical generated and could act as a better protectant compared to SbDHN1 or BSA under oxidative stress condition (Supplementary Figure S1B). The LDH activity was also affected by the FeSO_4_ or H_2_O_2_ alone. In presence of higher concentration of FeSO_4_ or H_2_O_2_, LDH activity was inhibited. For the experimental set up, combination of 0.1 mM FeSO_4_ and 1 mM H_2_O_2_ was chosen, as these were the minimum concentrations to inhibit the LDH activity more than 98%. However, using 0.1 mM FeSO_4_ and 1 mM H_2_O_2_ separately gave 26% and 32% inhibition of LDH activity, respectively (**Figures 1B,C**). SbDHN2 was also able to quench the effect of FeSO_4_ or H_2_O_2_ to a greater extent compared to SbDHN1. In presence of 0.1 mM FeSO_4_ the LDH can retain up to 74% of its activity. However, in presence of SbDHN1, SbDHN2 or BSA at a concentration of 1 μg/μl the enzyme showed 81, 100, and 75% of its activity, respectively. On the other hand in presence of 1 mM H_2_O_2_, LDH could retain 68% of its activity, however, in presence of SbDHN1, SbDHN2, or BSA at a concentration of 1 μg/μl, LDH showed approximately 87, 92, and 78% of its activity, respectively. Our result was further validated by in-gel analysis where in the presence of SbDHN2 protein, LDH was protected to a greater extent as compared to SbDHN1 or BSA when equal amount of protein was used in the assay (Supplementary Figure S2).

### Analysis of Transgenic Tobacco Plants

*SbDhn1* and *SbDhn2* transgenic plants were produced by *Agrobacterium-*mediated gene transformation method. Both the genes were transformed under the control of CaMV 35S promoter and Nos terminator. Generated transgenic lines were PCR checked for *Sbdhn1* or *Sbdhn2* and *hpt* gene. Six PCR positive lines were randomly selected (Line 1, 6, and 10 for *Sbdhn1* transformed plants and Line 4, 8, and 14 for *Sbdhn2* transformed plants). Transcript analysis was carried out for the transgenic NT and VT plants. The *SbDhn1* and *SbDhn2* transformed plants were positive for both *hpt* and dehydrin genes, respectively. However, the empty vector transformed lines showed amplification for *hpt* gene only. The NT tobacco plants did not show any amplification for either dehydrins or *hpt* genes (Supplementary Figure S3).

### Transgenic Tobacco Plants Overexpressing Dehydrin Genes Showed Better Protection under Oxidative Stress Conditions

**FIGURE 2:**
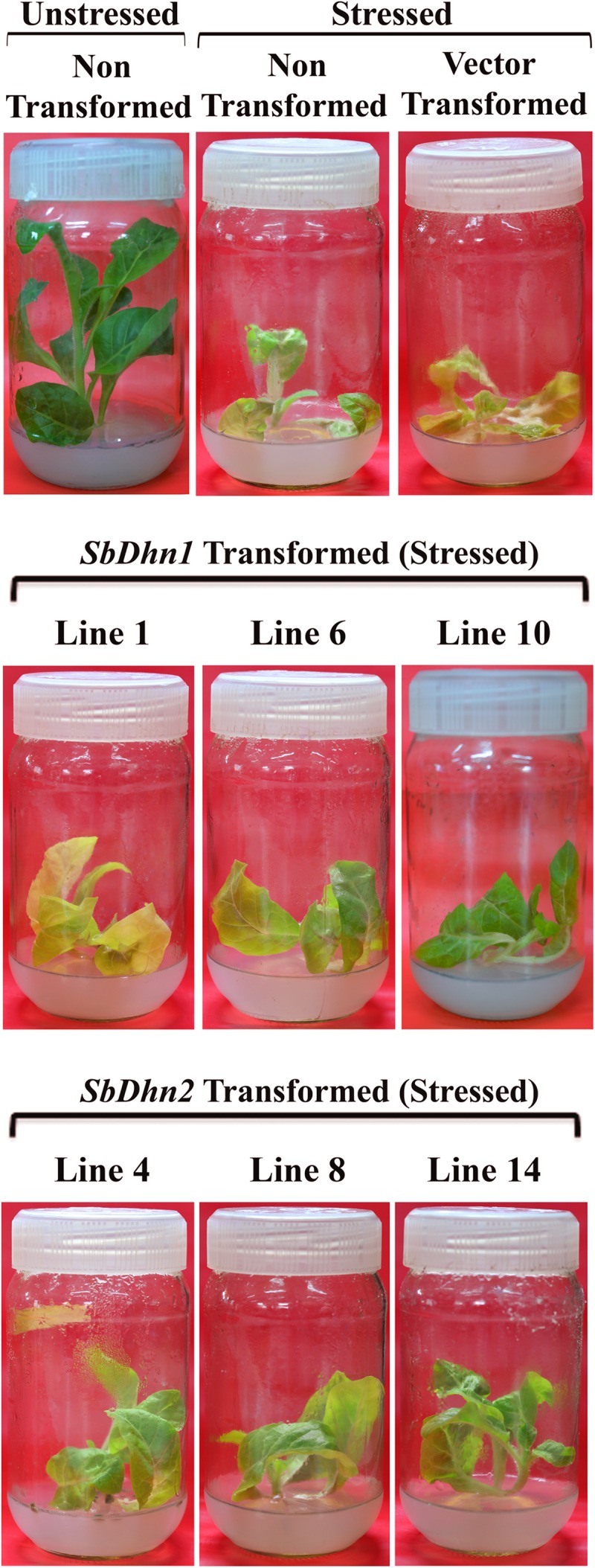
The transgenic tobacco lines (*SbDhn1* and *SbDhn2*) along with NT and VT plants grown in MS medium supplemented with 50 μM MV. NT tobacco plants grown under culture condition served as a control in this experiment. Transgenic lines showing better growth compared to NT and VT plants.

**FIGURE 3:**
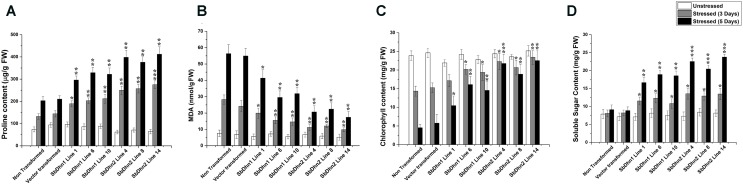
Measurements of different physiological parameters of transgenic tobacco plants (*SbDhn1* and *SbDhn2*) along with NT and VT plants after oxidative stress treatment induced by 50 μM of MV in MS medium. All the parameters were collected at 0 day, 3 days, and 5 days of stress treatment. **(A)** Proline content, **(B)** MDA content, **(C)** chlorophyll content, and **(D)** soluble sugar content. Error bar represents ± SEM from at least three experimental replicates. All data were statistically analyzed against stressed NT plants using Student’s *t*-test (^∗∗∗^*P* < 0.0005, ^∗∗^*P* < 0.005, and ^∗^*P* < 0.05). Data shown are illustrative of at least three independent experiments.

Leaf discs from NT, VT and transgenic lines over-expressing *Sbdhn1* and *Sbdhn2* genes were placed on half MS medium supplemented with 20 μM MV under continuous illumination for 5 days. Leaf disc from transformed lines over-expressing dehydrin genes showed the better tolerance as compared to NT or VT transformed plants under the stress situation. The NT and VT transformed leaf discs turned pale yellow in color. The leaf discs from *SbDhn1* and *SbDhn2* transformed plants showed better protection and remained green even after 5 days of MV treatment (Supplementary Figure S4). To validate this observation further, whole plant assay was performed where plants were placed on MS media supplemented with 50 μM MV. The transgenic lines expressing dehydrin genes showed better phenotypic expression compared to NT or VT plants (**Figure 2**) after 5 days of stress treatment. Proline is an important component of cell wall protein which protects membrane integrity and photosynthetic apparatus. (Rai et al., 2012; Ravikumar et al., 2014; Szabados and Savoure, 2010). The proline content increased significantly in transgenic lines overexpressing dehydrin genes. The proline content increased considerably in NT or VT tobacco plants when subjected to MV treatment as opposed to unstressed plants. The level of proline was more in *SbDhn2* plants as compared to *SbDhn1* plants when subjected to MV treatment. The level of proline accumulation was also higher when the plants were subjected to larger duration of stress treatment (**Figure 3A**). The MDA content increased considerably in the NT or VT plants as compared to that of transgenic lines. The MDA content increased in all the experimental setups under stress treatment when compared to unstressed control plants. Moreover the amount of MDA content increased with the duration of stress treatment (**Figure 3B**). Leaf chlorophyll content directly impacts the photosynthetic rate of plants (Ravikumar et al., 2014). ROS generated through the osmotic and oxidative stresses have detrimental effects on both the photosynthetic machinery as well as on the total chlorophyll content of the leaves. The chlorophyll content was also higher in plants over-expressing dehydrin genes, as compared to NT and VT plants when treated with MV for 3 and 5 days (**Figure 3C**). Under the stressed condition, the soluble sugar content present in the transformed plants over-expressing dehydrin genes was much higher as compared to NT or VT plants (**Figure 3D**).

### Photosynthetic Efficiency Analysis Showed Better Protection of the Over-expressed Dehydrin Lines

**FIGURE 4:**
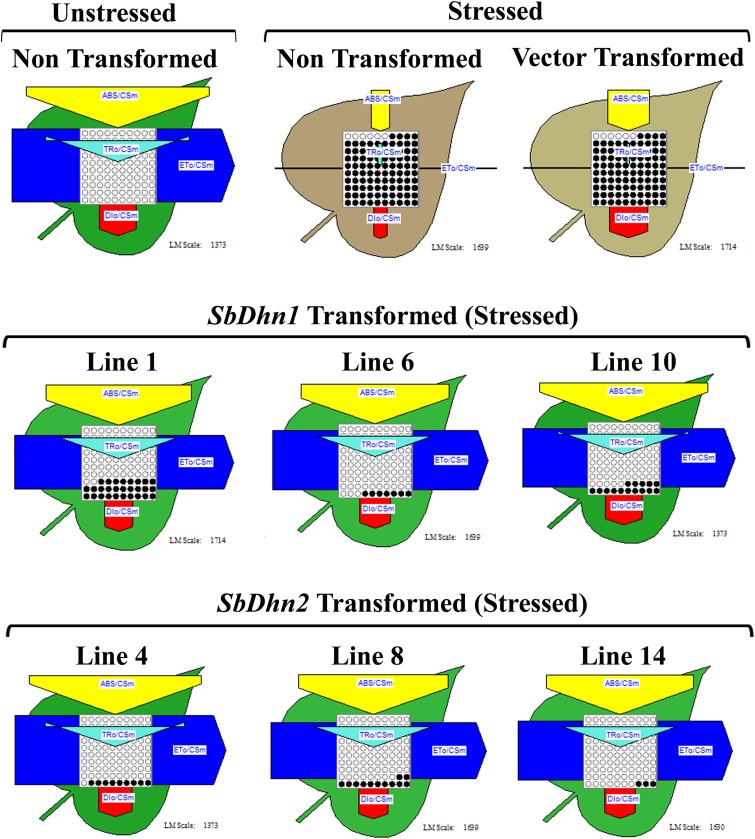
Analysis of photosynthetic efficiency of the NT, VT and transgenic lines over-expressing dehydrin gene(s) (*SbDhn1* and *SbDhn2*) after oxidative stress treatment by 50 μM MV for 5 days. Unstressed NT plants grown under normal growth condition served as a control in this experiment. Phenomenological yield models per exited cross-section under oxidative stress; yellow triangle, absorption maxima per excited cross-section (ABS/CS_m_); blue pipe, electron transport per excited cross-section (ET_0_/CS_m_); green triangle, trapped energy per excited cross-section (TR_0_/CS_m_); red block, dissipation maxima per excited cross-section (DI_0_/CS_m_); empty circles, active reaction centers; filled circles, inactive reaction centers.

The non-transformed plants which were kept under normal culture conditions showed fully functional electron transport chain as shown in **Figure 4**. However, after exposure to MV, the transformed plants over-expressing dehydrin genes showed a significantly higher level of photosynthetic efficiency than NT or VT plants as shown in **Figure 4**. The electron transport chain showed blocked reaction center in case of NT or VT plants while transgenic lines over-expressing dehydrin genes showed fully functional reactive center comparable to that of the unstressed plant even under oxidative stress condition.

### Dehydrins Protects the Photosynthetic Apparatus against Oxidative Damage

**FIGURE 5:**
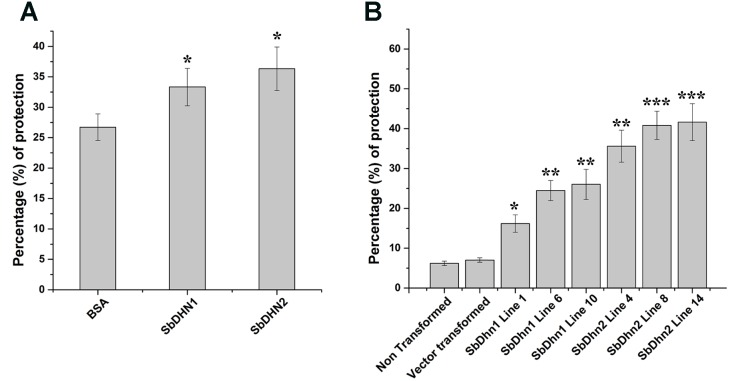
Effect of MV treatment on photosynthetic electron transport in intact chloroplast. **(A)** Percentage of protection exhibited by externally supplied dehydrin (SbDHN1 and SbDHN2) or BSA on photosynthetic electron transport in intact chloroplast in presence of MV. **(B)** Effect of MV treatment on photosynthetic electron transport in intact chloroplast isolated from transgenic tobacco plants (*SbDhn1* and *SbDhn2*) along with NT and VT plants. Error bar represents ± SEM from at least three experimental replicates. All data were statistically analyzed using Student’s *t*-test (^∗∗∗^*P* < 0.0005, ^∗∗^*P* < 0.005, and ^∗^*P* < 0.05). Data shown are illustrative of at least three independent experiments.

In order to analyze the effect of MV treatment on photosynthetic electron transport we isolated intact chloroplasts and subjected them to MV in presence or absence of dehydrins (SbDHN1 and SbDHN2) and kept under continuous illumination. MV acts as a terminal electron acceptor and generates superoxide radicals in presence of oxygen. Addition of MnCl_2_ which is a known scavenger of superoxide radicals prior to addition of MV and its incubation in presence of light showed reduction of DCPIP (data not shown). However, when only intact chloroplasts were incubated with MV in presence of light it showed little reduction of DCPIP as the superoxide radicals generated in the process might have disrupted the electron transport chain considerably. High amount of ROS generated leads to membrane rupture. BSA was used as a control in this experiment. The results showed that the dehydrins were able to render protection to a better extent as compared to BSA (**Figure 5A**). While the protection activity was better for SbDHN2, but SbDHN1 also provided a fair amount of protection as compared to BSA. Similar result was obtained when chloroplasts isolated from transgenic plants were subjected to MV treatment (**Figure 5B**). The membrane association property of dehydrin might be one explanation how dehydrin protects the chloroplast membrane and subsequently the electron transport chain.

### Scavenging of Superoxide Radicals Impart Protection to Transgenic Plants

**FIGURE 6:**
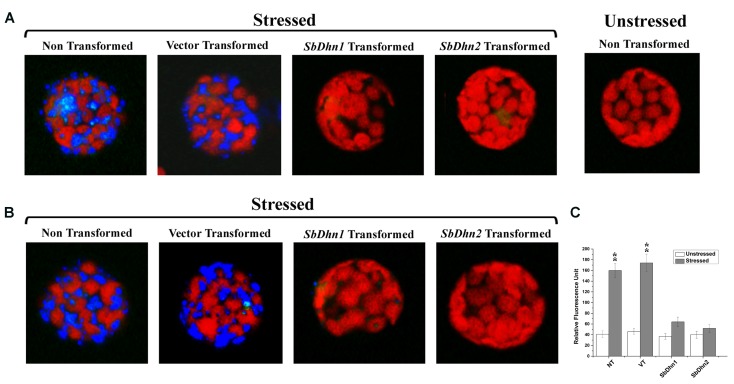
Fluorescence microscope images of isolated protoplast from tobacco plants stained with DHE **(A)** Protoplast isolated from of NT, VT, and transgenic lines over expressing dehydrin genes (*SbDhn1* and *SbDhn2*) and then subjected to MV treatment followed by staining with DHE **(B)** Intact protoplast was isolated from NT, VT and transgenic lines over expressing dehydrin genes (*SbDhn1* and *SbDhn2*) subjected to 50 μM MV for 5 days. In both the cases protoplast isolated from NT plants without any stress treatment served as control. **(C)** The relative fluorescence obtained after disruption of the intact protoplast isolated from NT, VT and transgenic lines (*SbDhn1* and *SbDhn2*) subjected to 0 μM (unstressed) and 50 μM (stressed) MV for 5 days and stained with DHE. Error bar represents ± SEM from at least three experimental replicates. All data were statistically analyzed using Student’s *t*-test (^∗∗^*P* < 0.005 and ^∗^*P* < 0.05). Data shown are illustrative of at least three independent experiments.

Reactive oxygen species like superoxide radicals were generated as a result of oxidative stress. DHE was used as a probe for *in situ* localization of the superoxide radical generated under oxidative stress treatment. DHE produces a blue fluorescence until oxidized to ethidium. The superoxide radicals generated in the isolated protoplasts from NT, VT and transgenic lines over expressing dehydrin gene(s) after treatment with 10 mM MV were clearly visible under fluorescence microscope as a blue fluorescence. The level of super oxide generation was very high in protoplast isolated from NT and VT plants as compared to *SbDhn1* and *SbDhn2* transgenic plants (**Figure 6A**). The occurrence of the dehydrin proteins in the cytoplasm might scavenge the superoxide radicals generated during oxidative stress.

Similar result was obtained when the whole plants were also treated with MV and the leaves were collected after 5 days of stress treatment. The superoxide radicals generated in NT plants under normal growth conditions was lower as compared to MV treated plants. The transformed plants over-expressing dehydrin genes showed a very low level of blue fluorescence in case of *SbDhn1* over-expressed plants when subjected to MV treatment. However, the blue fluorescence was not detected in *SbDhn2* transformed plants indicating the superoxide radicals generated was even less in plants transformed with *SbDhn2* (**Figure 6B**). Similar data was obtained in fluorescence spectrometer analysis; the relative fluorescence was much higher in the NT and VT plants than transgenic line after oxidative stress treatments (**Figure 6C**).

### Transformed Line Overexpressing Dehydrin Genes Showed Low Level of H_2_O_2_

**FIGURE 7:**
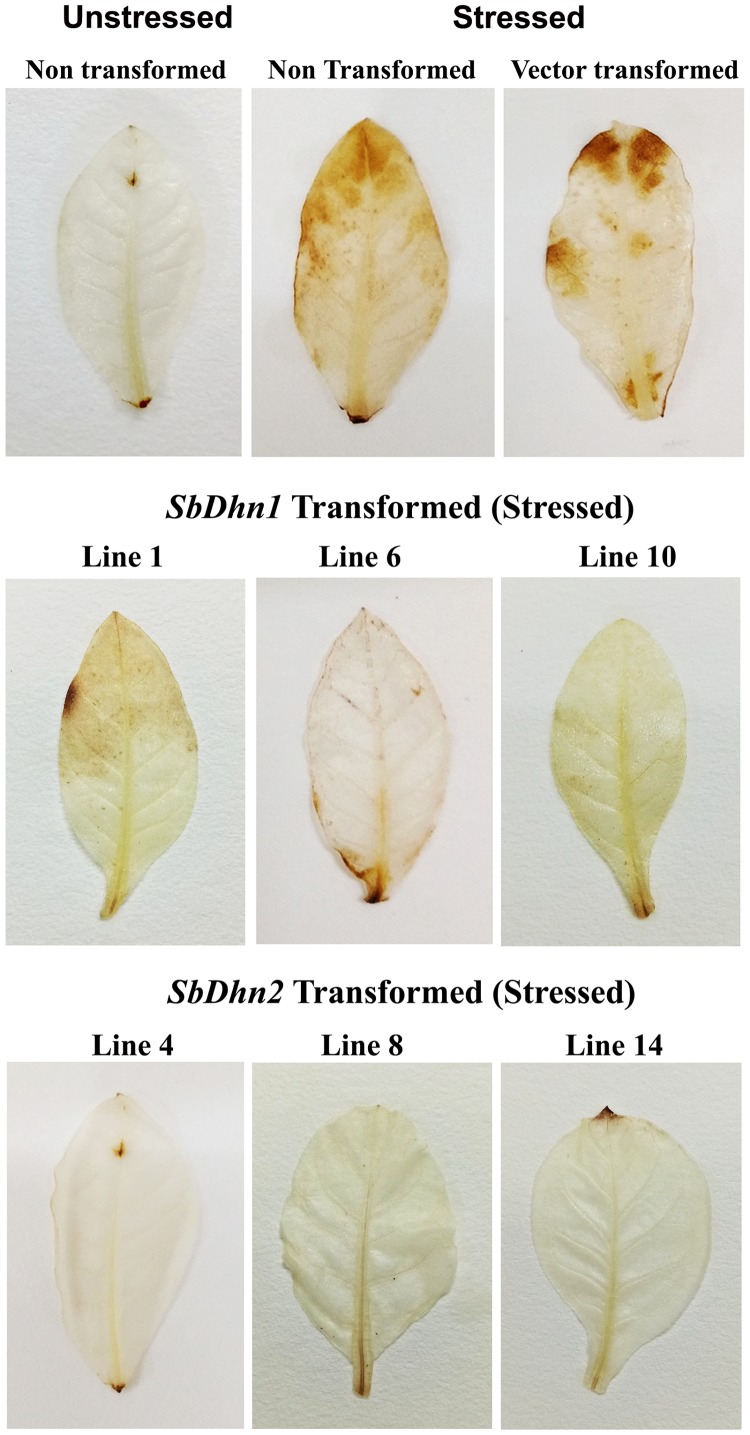
Accumulation of H_2_O_2_ in the leaves of NT, VT and transgenic lines (*SbDhn1* and *SbDhn2*) after oxidative stress imparted by 50 μM MV, as revealed by staining with DAB.

The low level of superoxide radicals generated in transgenic lines implies that the level of H_2_O_2_ formed should also be less in the transgenic plants. After the stress treatment with MV, H_2_O_2_ accumulation in NT and VT plants was found to be higher, with respect to the dehydrin overexpressing transgenic plants. DAB stained leaves after subsequent removal of chlorophyll showed brown patches of H_2_O_2_ accumulation in the leaf tissues of NT and VT plants. The *SbDhn2* plants showed lower level of H_2_O_2_ accumulation even compared to *SbDhn1* plants (**Figure 7**).

### The Low Level of ROS in Plants Over-expressing Dehydrin Genes Positively Correlates with an Increase in Enzymatic Activity

**FIGURE 8:**
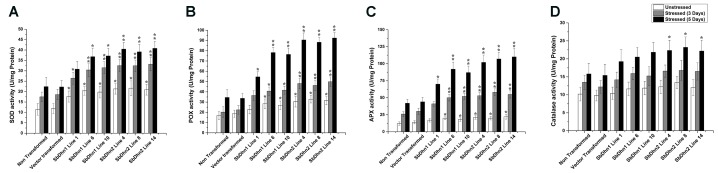
Measurements of antioxidant enzyme activity of transgenic tobacco plants along with NT and VT plants after oxidative stress treatment generated by 50 μM of MV in MS medium. Enzyme activities were measured at 0 day, 3 days, and 5 days of stress treatment. **(A)** SOD, **(B)** POX, **(C)** APX, and **(D)** CAT. Error bar represents ± SEM from at least three experimental replicates. All data were statistically analyzed against stressed wild plants using Student’s *t*-test (^∗∗^*P* < 0.005 and ^∗^*P* < 0.05). Data shown are illustrative of at least three independent experiments.

The low level of ROS generated in transgenic lines implies that the level of the ROS scavenging enzymes will also be increased. In order to validate this, we determined the enzymatic activity for SOD, CAT, POX, and APX in NT and transgenic lines following treatment with 50 μM of MV at an interval of 0 day (unstressed), 3 days, and 5 days. The activity levels of SOD, POX, and APX increased significantly in transgenic lines over-expressing dehydrin genes as compared to NT or VT plants after the stress treatment (**Figures 8A–C**). However, the CAT activity showed little change compared to other ROS scavenging enzymes. The only significant change was observed in case of *SbDhn2* over-expressing line after 5 days of stress treatment (**Figure 8D**). Though, the enzyme activities for all the enzymes studied here increased with the duration of the stress treatment in all the experimental lines when subjected to MV treatment, the activity was higher for transformed plants over-expressing dehydrin genes (both *Sbdhn1* and *Sbdhn2*). The qRT-PCR analysis also showed an upregulation of all the enzymes after stress treatment compared to unstressed control. However, the transcript level of the enzymes studied here (SOD, POX, APX, and CAT genes) were higher in the transgenic lines compared to that of NT and VT plants after MV-induced oxidative stress treatment (Supplementary Figure S5).

## Discussion

Generation of ROS during oxidative conditions leads to damaging effect to the membranes and biomolecules. ROS plays an important role in signaling system in plants and controls processes like growth and development. It acts as a signal in both biotic and abiotic environmental cues (Apel and Hirt, 2004). The ROS includes free radicals like 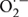, OH^⋅^ and non-radicals like H_2_O_2_ and ^1^O_2_. Although in low concentration it can act as a signaling molecule, an increased level of this can induce oxidative damage. The delicate balance between production and mitigation of ROS, therefore, remains exceedingly important. The hydroxyl radicals are more reactive compared to the superoxides and H_2_O_2_, thereby readily attacking the lipids, nucleic acids, and proteins at, or close to, the site of their formation (Halliwell and Gutteridge, 1990; Asada, 1994). Therefore, protection towards these highly reactive hydroxyl radicals can be achieved by a mechanism that can prevent its formation. But once produced, the protection depends entirely on the presence of radical scavengers and antioxidants in the close vicinity. The hydroxyl radicals generated *in vivo* originate mainly from the decomposition of H_2_O_2_, in presence of metal ions such as Fe^2+^ and Cu^2+^ where superoxide radicals react with Fe^3+^to form Fe^2+^ and O_2_ in the process (Asada, 1994; Allen, 1995).

$$H_{2}O_{2}+O_{2}^{\cdot -}\to {OH}^{-}+O_{2}+OH\cdot$$

The generation of the highly reactive hydroxyl radicals depend on the superoxide radicals, H_2_O_2_ and transition metal ions. Scavenging any or all of these components responsible for hydroxyl radical generation will inhibit its formation. In this report, we provided direct evidence to show the protective role of dehydrins in retaining LDH activity in presence of H_2_O_2_ and Fe^2+^. The better performance of SbDHN2 compared to the SbDHN1 could be attributed to the fact that SbDHN2 could sequester metal ions owing to a large number of histidine residues present in the amino acid sequence as has been reported earlier by Halder et al. (2016) (Supplementary Table S2). Low level of protection of LDH activity was also observed when BSA was used as a protein control, suggesting that the amino acids and peptide bonds provide a fair amount of protection of LDH activity under oxidative stress condition.

In order to determine if dehydrin can scavenge the free radicals and impart protection against the highly reactive hydroxyl radicals *in vivo*, we transformed the wild-type tobacco plants with dehydrin gene(s) from Sorghum. The components for generation of hydroxyl radicals were abundant in the chloroplasts because superoxide radicals and H_2_O_2_ are produced in the Mehler reaction of PSI. The generation of hydroxyl radicals lead to damaging conditions for different cellular components by way of LPO thereby leading to disruption of membranes and damaging other biomolecules. Since no single enzymatic system is available for destroying this most reactive radical, therefore excess accumulation of OH^⋅^ leads to cellular death (Pinto et al., 2003).

One of the primary components for generation of hydroxyl radical is the superoxide radicals; therefore scavenging off these ROS can, in turn inhibit the production of hydroxyl radical. The MV treatment of intact chloroplasts isolated from NT, VT and transgenic lines overexpressing dehydrin genes was carried out. MV acts as a terminal electron acceptor and subsequently gets oxidized generating superoxide radicals. The increased superoxide radicals can lead to membrane damage thus ultimately inhibiting the electron transport chain. The membrane association property of dehydrin provides a certain level of protection to the isolated chloroplast. The occurrence of K-segment in the dehydrin renders protection against oxidative damage as reported earlier (Liu et al., 2017) possibly by “shielding effect.” Additionally, the number and composition of amino acids present in dehydrin might also play an additive role in such protection as large number of histidine residues could sequester the transition metal ions making them unavailable for Fenton reaction. The shielding effect exhibited by dehydrins might be one explanation for such protection. Our results proved beyond doubt that the level of superoxides generated in transgenic lines were significantly lower as compared to NT or VT plants subjected to same stress treatment. These results were further substantiated by the presence of low level of superoxides in the protoplasts isolated from transgenic plants compared with NT and VT plants as evident by the fluorescence level observed with DHE as a probe. The intracellular superoxide radical generation was detected with DHE when excited at 396 nm allowing more selective imaging of the hydroxylated products produced by superoxide radicals. The fluorescence increased in NT tobacco plants when treated with MV that generates superoxide radicals under illuminated condition. However, the fluorescence intensity generated in over-expressing dehydrin lines was significantly lower as compared with NT or VT lines upon treatment with MV.

An increase in the free proline content was also evident in transgenic lines over-expressing dehydrin genes when compared with WT and VT plants. Apart from acting as osmoprotectant, proline also serves as a scavenger of the free radical thus protecting the biomolecules against oxidative damage (Smirnoff and Cumbes, 1989; Saradhi and Mohanty, 1993; Kaul et al., 2008). The increased proline and other antioxidants at the site of formation of OH^⋅^ might help to ameliorate the OH^⋅^ with high reactivity and low half-life (Signorelli et al., 2013). Though certain amino acids are capable of scavenging the superoxide radicals; however, the large number of proteins present in a cell remains inadequate in scavenging the superoxide radicals which seems to be a property associated with dehydrin. ROS produced in plants under oxidative stress conditions leads to higher MDA content produced due to lipid peroxidation of cell membranes. Our results showed lower levels of MDA in the transgenic lines when compared with WT and VT plants under oxidative stress conditions. The chlorophyll and soluble sugar content were higher in transgenic lines implying that dehydrin over expression results in protection of the photosynthetic machinery in the chloroplast. Furthermore, transgenic plants showed lower levels of H_2_O_2_ when compared with the control plants under stress treatment, as evident by DAB staining of the leaves.

The dismutation of superoxide radicals by SOD also played a significant role. SOD is primarily an important enzyme acting in detoxification reaction which can effectively convert the O_2⋅_ radicals to H_2_O_2_. The rate of ROS production increases significantly during stress conditions. Detoxification of the increased ROS is effectively carried out by the ROS-scavenging enzymes, namely CAT, SOD, APX, and POXs (Mittler et al., 2004). Additionally, the enhanced activity of the enzyme SOD in the transgenic lines overexpressing dehydrin gene(s) as compared to NT or VT plants might be the result of an indirect protective effect of dehydrin genes. An increase in SOD activity was documented in the leaf tissue obtained from transgenic dehydrin lines as compared to the zero time point measurement under stress treatment. Correspondingly an increase in the activity level of other antioxidant enzymes like APX and POX was also observed. Increase in activity of other antioxidant enzymes suggests that, after dismutation of superoxide radicals into H_2_O_2_ and O_2_ by SOD, APX reduces H_2_O_2_ into the water in presence of ascorbic acid. Increased APX may constitute a protection mechanism against H_2_O_2_. CAT also plays a significant role in maintaining the ROS homeostasis by catalyzing the decomposition of H_2_O_2_ (Du et al., 2008). However, the CAT activity showed only a small level of increase in its activity. This might be due to the fact that CAT activity depends on the increased H_2_O_2_ concentration and a certain threshold level of H_2_O_2_ perhaps is essential for CAT activity. The localized occurrence of CAT in peroxisomes and a very low affinity for H_2_O_2_ might be the reason for its small change in activity level. Our results strongly suggest that functional co-operation between the radical scavenging enzymes remains essential for protection against the ROS generated.

There are substantial evidences documented here that dehydrins can scavenge the superoxide radicals generated thereby increasing the ability to cope with the oxidative damage generated as a result of MV treatment. An increase in ROS scavenging enzymes might function cooperatively with the dehydrins but the actual mechanism how this happens remains unexplained. Scavenging of superoxide radicals and transition metals by SbDHN2 led to a better protection of plants as compared to SbDHN1. Thus we conclude from the evidences documented from *in vitro* and *in vivo* experiments that there lies an indirect involvement of dehydrin in protection against hydroxyl radical. Therefore over-expression of dehydrins have resulted in an increased resistance to oxidative damage which is reflected by the better photosynthetic efficiency of the transgenic plants as compared to NT or VT plants. Increase in soluble sugars, radical scavengers, enzymes related to antioxidative pathways, sequestration of transition metal ions all allow for an ROS homeostasis inside the cells.

## Author Contributions

All authors have participated sufficiently in the work to take public responsibility for appropriate portions of the content. No one, other than the authors listed below has contributed substantially to the writing and revising of the manuscript. Contributors who do not meet the criteria for authorship have been listed in the acknowledgment. Study conception and design: SR, TH, and GU; Acquisition of data: TH, GU, CB, AD, and CC; Analysis and interpretation of data: TH, GU, CB, AD, and CC; Drafting of manuscript: SR, TH, and GU; Critical revision: SR.

## Conflict of Interest Statement

The authors declare that the research was conducted in the absence of any commercial or financial relationships that could be construed as a potential conflict of interest.

## Abbreviations

APX: ascorbate peroxidase
BAP: 6-benzylaminopurine
BSA: bovine serum albumin
CAT: catalase
CIB: chloroplast isolation buffer
DAB: 3,3′-diaminobenzidine
DCPIP: 2,6-dichlorophenolindophenol
DHE: dihydroethidium
Fv/Fm: maximum quantum yield of PSII
H_2_O_2_: hydrogen peroxide
LDH: lactate dehydrogenase
LEA: late embryogenesis abundant
MDA: malonyl dialdehyde
MS: Murashige and Skoog
MV: methyl viologen
NAA: naphthalene acetic acid
NT: non-transformed
^1^O_2_: singlet oxygen
superoxide radical
OH^⋅^: hydroxyl radical
PCR: polymerase chain reaction
POX: peroxidase
ROS: reactive oxygen species
SDS-PAGE: sodium sodecyl sulfate-polyacrylamide gel electrophoresis
SOD: superoxide dismutase
TBA: thiobarbituric acid
TCA: trichloroacetic acid
VT: vector transformed
