# Supplementary material for: Dehydrins Impart Protection against Oxidative Stress in Transgenic Tobacco Plants
Source: Front Plant Sci. 2018 Feb 14;9:136. doi: 10.3389/fpls.2018.00136 (PMC5817096; doi:10.3389/fpls.2018.00136)
Supplement: Supplementary file 1 [file Data_Sheet_1.PDF]

Supplementary Table S1: List of primers used

Name of the gene	Sequence of the oligo(s) used
Dehydrin (*SbDhn1*)	5′ *CATATG*GAGTACGGTCAGCAGGGACAGCAC 3′
	5′ *CTCGAG*GTGCTGTCCGGGCAGCTTCTCCTTG 3′
Dehydrin (*SbDhn2*)	5′ *CATATG*GAGGATGAGAGGAGCACCCAGCAG 3′
	5′ *CTCGAG*GGAGCTGGTCTTGTGCTCGCCG 3′
Hygromycin (*hpt*)	5′ ATGAAAAAGCCTGAACTCACCGCGAC 3′
	5′ TTCCTTTGCCCTCGGACGAGTGCTG 3′
Catalase (*Cat*)	5′ GGAAGGTTATGGTGTTCACG 3′
	5′ GCGTGGCTATGATTTGTACC 3′
Peroxidase (*Pox*)	5′ AGAGAATGTATGCCCTGGTG 3′
	5′ CTCCAGATCGGTTTGCTGT 3′
Ascorbate peroxidise (*Apx*)	5′ CCCACTGTAAGCGAGGAGTA 3′
	5′ CAAGACGGAGCATAAGAGGA 3′
Superoxide dismutase (*Sod*)	5′ GACTTCATGGCTTCCATGTC 3′
	5′ CTCATCTTCAGGAGCACCAT 3′
Actin (*Act*)	5′ GCAACTGGGATGATATGGAG 3′
	5′ TAGCCTTCGGGTTAAGAGGT 3′

Supplementary Table S2: Comparison of SbDHN1 and SbDHN2 protein

	**SbDHN1**	**SbDHN2**	**BSA**
**Size of protein**	152aa	283aa	607aa
**No. of Histidine Residues**	9	26	17
**Percentage of Histidine Residues**	(5.9%)	(9.2%)	(2.8%)

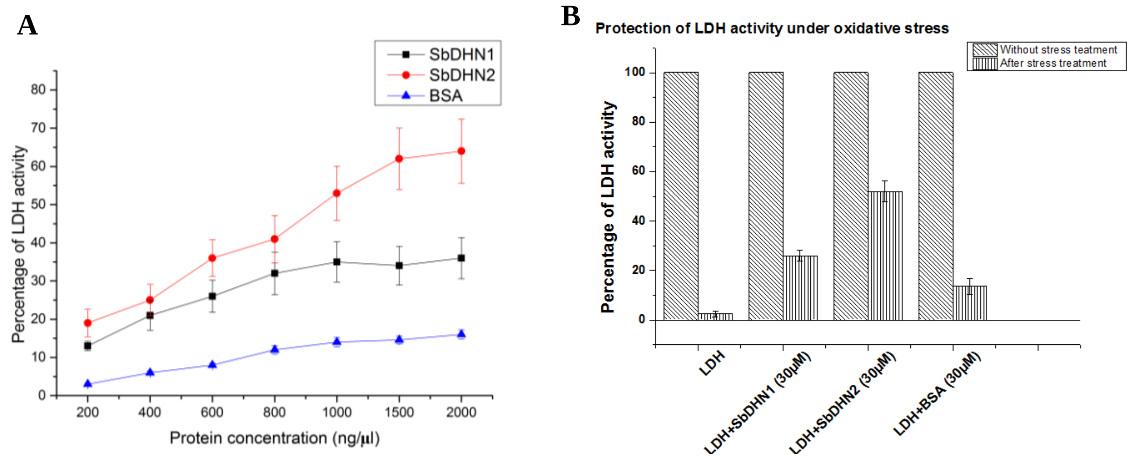

Supplementary Figure S1: Percentage of LDH activity under oxidative stress in presence of (A) different concentration of SbDHN1, SbDHN2 and BSA; and (B) equimolar concentration (30µM) of SbDHN1, SbDHN2 and BSA.

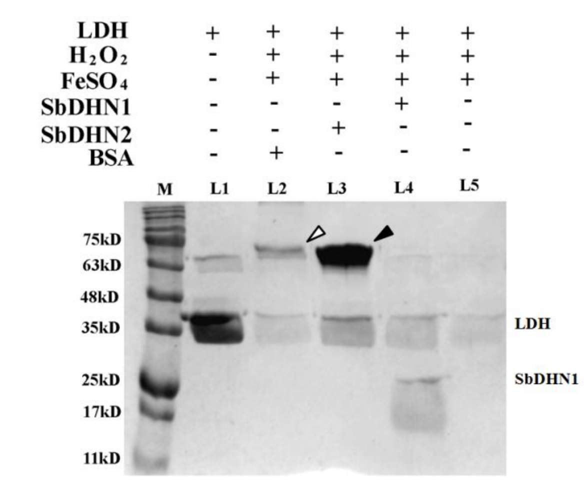

Supplementary Figure S2: SDS-PAGE showing the LDH protein after oxidative stress treatment in presence of SbDHN1, SbDHN2 (indicated by closed arrow◀) and BSA (indicated by open arrow◁).

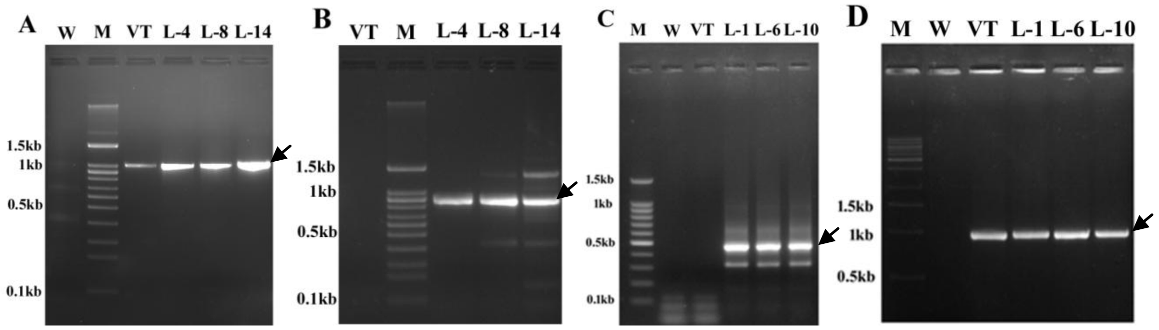

Supplementary Figure S3: Screening of the transgenic plants. **(A-B)** PCR amplification of *hpt* and *SbDhn2* genes from the putative transgenic lines (L-4, L-8 and L-14) along with NT (W) and VT **(C-D)** PCR amplification of *SbDhn1*and *hpt* genes from the putative transgenic lines (L-1, L-6 and L-10) along with NT (W) and VT .

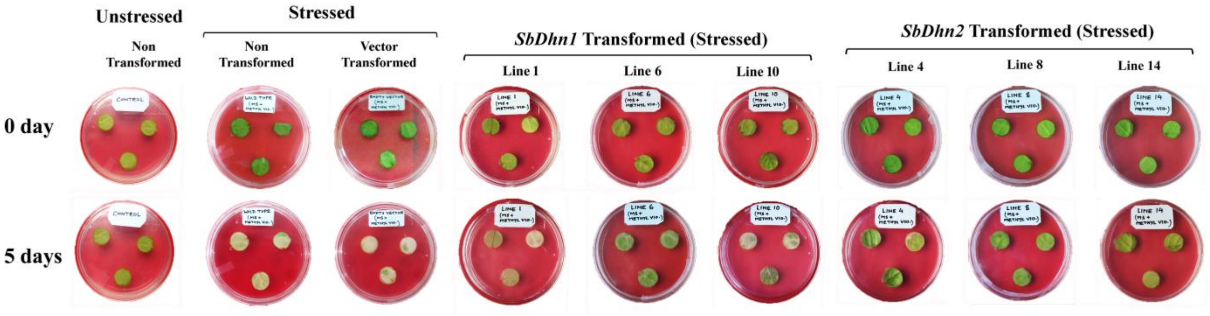

Supplementary Figure S4: Effect of oxidative stress on leaf discs of tobacco plants. Effect of Methyl viologen on leaf discs isolated from NT, VT and *SbDhn1* and *SbDhn2* transgenic lines at 0 day and after 5 days of incubation.

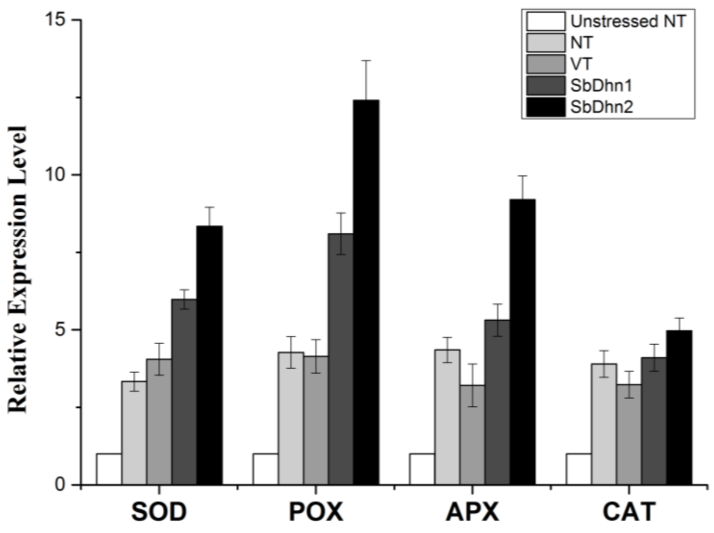

Supplementary Figure S5: Relative expression of the ROS scavenging enzymes after oxidative stress treatments.
